# Probabilistic tuning of attentional templates: Evidence for flexible prioritization in visual working memory

**DOI:** 10.1167/jov.26.8.6

**Published:** 2026-08-21

**Authors:** Alexandre Fortuna, Martin Constant, Dirk Kerzel

**Affiliations:** 1 Faculty of Psychology and Educational Sciences, University of Geneva, Switzerland

**Keywords:** visual search, attentional template, retro-cues, visual working memory

## Abstract

Attentional templates in visual working memory (VWM) are essential for guiding visual search. Although much debate has focused on whether these templates are stored in discrete states or continuous resources, a question remains: To what extent can the priority of a template be strategically tuned to match environmental probabilities? We investigated this flexibility by manipulating the reliability of retro-cues (the probability that a cued item is the target) across three experiments combining memory recall and visual search. Crucially, we tested whether strategic prioritization persists under varying memory loads (two vs. three items). We observed that retro-cue validity affected both search efficiency and memory performance and that these effects were larger under higher cue reliability. This reliability-dependent pattern was clearly present when memory load increased to three items (Experiment 2) and was also observed when item shape signaled the functional role of each representation (Experiment 3). Together, these results show that the influence of a cued representation varies systematically with cue reliability. This pattern is consistent with flexible prioritization but does not distinguish between a continuous adjustment of template strength and probabilistic switching between discrete representational states. Overall, our findings suggest that VWM prioritization is sensitive to expected relevance and adapts to memory load and task structure.

## Introduction

People devote a significant part of their daily lives to searching for familiar objects in complex, densely packed visual scenes. A common example is preparing dinner, where we often search for several specific ingredients in a cluttered fridge. Visual working memory (VWM) plays a key role in both perceptual attention and action control: It maintains target templates to guide perceptual selection and stores task sets that support goal-directed behavior (Myers, Stokes, & Nobre, 2017). In these cases, stored feature representations serve to guide attentional selection during visual search (e.g., the shape of an eggplant and the color of a tomato), known as attentional templates (Duncan & Humphreys, 1989) or attentional control sets (Folk, Remington, & Johnston, 1992). An attentional template is a mental representation of one or more target features used to guide visual search.

Electrophysiological studies have confirmed that templates are activated in VWM to control top–down selection (Carlisle, Arita, Pardo, & Woodman, 2011; Grubert & Eimer, 2018), but a critical functional question remains: How are these templates maintained and prioritized when memory resources are limited? Storing multiple items introduces competition, which can constrain attentional guidance (Carlisle & Woodman, 2019). This raises the question of whether the visual system manages this competition through rigid binary states or through a more flexible, graded allocation of priority.

In VWM research, retro-cues have long been used to show that attention can be reoriented toward internal representations in VWM (Souza & Oberauer, 2016) notably by strengthening weakly encoded representations during retention (Vandenbroucke, Sligte, & Lamme, 2011). Further, Berryhill, Richmond, Shay, and Olson (2012) showed that retro-cue benefits with arrow cues emerged even when they were non-predictive, suggesting that attentional orienting within VWM is not fully under strategic control.

At the same time, how multiple attentional templates are maintained and used in VWM remains unclear. Olivers, Peters, Houtkamp, and Roelfsema (2011) proposed the single-item-template hypothesis, which suggests that VWM operates with two representational states: an active state and an accessory state. In the active state, the item representation serves as an attentional template. In this framework, only a single representation at a time can be kept in an active state. All other items are maintained in an accessory state, where they cannot interact with visual search. When searching for multiple items, representations sequentially switch from the accessory state to the active state.

The single-item-template account has been developed largely on the basis of dual-task studies combining memory maintenance with visual search (e.g., Downing & Dodds, 2004; Olivers, Meijer, & Theeuwes, 2006; Soto, Hodsoll, Rotshtein, & Humphreys, 2008; Woodman & Luck, 2007; see also Olivers et al., 2011). In these tasks, participants keep an item in VWM while searching for a separate target. Although the memorized item is not relevant for the search itself, search performance is often more disrupted when a salient distractor shares features with that stored representation. This pattern has been taken to suggest that even non-prioritized contents of working memory (WM) can bias attentional selection.

However, memory-based interference has been reported primarily in paradigms where the search target remained stable across blocks (Kim & Cho, 2016; Olivers et al., 2006; Soto et al., 2008; van Moorselaar, Theeuwes, & Olivers, 2014). Under these conditions, the attentional template can be offloaded to long-term memory, freeing VWM for the accessory representation to become active and interfere with search. By contrast, when the target changes from one trial to the next, the attentional template is constantly updated in VWM and must therefore remain in the active state. As a result, the other representation remains accessory and does not produce memory-based interference (Downing & Dodds, 2004; Hollingworth & Hwang, 2013; Olivers, 2009; Woodman & Luck, 2007).

Although the single-item-template hypothesis has received considerable support (Olivers et al., 2011; van Moorselaar et al., 2014), several elements call it into question. Initial evidence that multiple items could guide attention at the same time was provided by Beck, Hollingworth, and Luck (2012). They showed that a participant's gaze alternated frequently between items matching two attentional templates without the switch cost that would be predicted if only a single attentional template was used. This indicated that both WM representations could guide attention concurrently. This has been further demonstrated in several subsequent behavioral and electrophysiological studies (Berggren, Nako, & Eimer, 2020; Carlisle & Woodman, 2019; Grubert & Eimer, 2015; Grubert & Eimer, 2016; Hollingworth & Beck, 2016; Zhou, Lorist, & Mathôt, 2020). At the same time, this influence may depend on the relative functional status or priority of the representations in VWM (Carlisle & Woodman, 2019; Kerzel & Witzel, 2019). These findings gave rise to the multiple-item-template hypothesis, which assumes that several items stored in VWM can concurrently serve as attentional templates, allowing attention to be directed by multiple targets simultaneously.

Frătescu, Van Moorselaar, and Mathôt (2019) conducted replications of the studies by van Moorselaar et al. (2014) and Hollingworth and Beck (2016), which provide evidence for the single- and multiple-item-template theories, respectively. Interestingly, they successfully replicated both studies despite their mutually exclusive underlying theories. This demonstrates that both theories should be further refined. One possible way to reconcile these findings is to assume flexible prioritization within VWM, whereby attentional priority can be allocated to one or several memory representations depending on task demands.

Evidence for flexible prioritization also comes from memory recall tasks. For example, Gunseli, van Moorselaar, Meeter, and Olivers (2015) showed that cue reliability influenced how strongly participants prioritized the cued representation during maintenance. The validity effects were larger in the high-reliability condition than in the low-reliability condition. The authors interpreted this dissociation as consistent with models distinguishing between items in the active state and other representations maintained in an accessory state. However, whether such reliability-dependent prioritization also extends to attentional guidance during visual search remains unclear.

Using a dual-task paradigm, Dube and Al-Aidroos (2019) tested whether a memory-matching distractor would capture attention during visual search following deterministic versus probabilistic (100% valid or not, respectively) retro-cues. Although probabilistic retro-cues improved memory performance, they did not produce attentional capture during visual search. Attentional capture was observed only with deterministic cues. Based on this pattern, the authors interpreted their findings as consistent with the single-item-template account: A memory representation guides search only after entering an active guiding state, rather than varying gradually with cue reliability. Yet, it remains possible that this pattern was partly related to the relatively low memory load used in that paradigm. When few items are maintained, competition within VWM may be limited, potentially reducing the observable impact of cue reliability on attentional guidance (Souza & Oberauer, 2016). In the present study, we therefore assessed cue-reliability effects first under low memory load in Experiment 1 and then examined whether the same pattern would hold when memory load was increased in Experiment 2.

Here, we investigated whether retro-cues modulate the priority of attentional templates in an all-or-none mechanism or whether it is dependent on their reliability. Although prior research has established that retro-cues can improve recall accuracy, it remains an open question whether the reliability of these cues can modulate attentional efficiency. Clarifying this point would help determine whether cue reliability shapes both memory and search behavior in a similar way, or whether these functions dissociate.

In three preregistered experiments, participants remembered colors and then performed either a visual search task (guiding template) or a continuous-recall task (target template). Visual search performance (response times and accuracy) was used to assess how effectively maintained representations supported attentional guidance, whereas continuous recall precision was used to assess the fidelity of those representations for later memory report. Experiment 1 examined whether manipulating retro-cue reliability produces graded prioritization effects on memory precision and search performance under low memory load (two items), and Experiment 2 increased this load to three items to test whether these effects can be elicited with stronger resource competition.

Finally, Experiment 3 addressed the role of task structure. In the first two experiments, participants did not know during maintenance which memorized item would later be relevant for visual search or for memory recall, which may have encouraged a more general maintenance strategy. Experiment 3 introduced specific shape–task associations, such that each item had a stable functional role. This manipulation allowed us to test whether predefined shape-task association facilitates resource allocation while uncertainty about the upcoming task remained.

## Experiment 1

The aim of Experiment 1 was to test whether the prioritization of attentional templates varies in a graded manner as a function of cue certainty under low WM load. By presenting only two items, we created a situation of mild resource competition to isolate this tuning mechanism. Specifically, we tested whether increasing the predictive utility of the retro-cue leads to a gradual reallocation of priority, resulting in improved memory precision and better visual search performance. We assumed that response times (RTs) in the search task and recall accuracy in the memory task would depend on the priority assigned to the cued stimulus. We therefore expected better performance following valid than invalid retro-cues.

Crucially, we expected the magnitude of this validity effect to vary with retro-cue reliability. This prediction is consistent with resource-based accounts in which priority modulates the allocation of limited resources, thereby influencing memory precision (Bays et al., 2009) and the effectiveness of attentional guidance during visual search (Huynh Cong & Kerzel, 2021; Souza & Oberauer, 2016). More specifically, we expected the magnitude of the validity effect to scale with retro-cue reliability. When the cue is uninformative (i.e., 50% reliability), participants should ideally ignore it. We therefore expected no difference between valid and invalid trials in this condition. Any residual validity effect in this condition therefore reflects non-strategic factors, such as automatic orienting or unsystematic variability, rather than deliberate resource allocation. By contrast, when the cue carries predictive value (here, 70% reliability), the allocation of resources should follow this predictive utility. Under these conditions, the system should tune the weights of the representations more sharply, allocating more resources to the likely target.

### Methods

#### Transparency and openness

All experiments were preregistered at the following links: Experiment 1 (https://osf.io/tgbdc), Experiment 2 (https://osf.io/jnwvt), and Experiment 3 (https://osf.io/js9yp). The cleaned data, experimental scripts, analysis code, and colors used are available at: https://osf.io/q8d47/ (Fortuna, Constant, & Kerzel, 2024). Data were analyzed using CPython 3.12.4 with the following packages: *numpy* 2.0.0 (Harris et al., 2020), *seaborn* 0.13.2 (Waskom, 2021), *matplotlib* 3.9.1 (Hunter, 2007), *pingouin* 0.5.4 (for the repeated-measures analyses of variance [ANOVAs]) (Vallat, 2018), and *pandas* 2.2.3 (The pandas Development Team, 2025). The experiment was programmed with PsychoPy 2024.2.1 (Peirce et al., 2019). Post hoc analyses were conducted using R 4.5.0 (R Core Team, 2026) with the *effectsize* 1.00 package (Ben-Shachar, Lüdecke, & Makowski, 2020). Chronologically, we conducted Experiment 1 first, then Experiment 3 and finally Experiment 2. Experiments 1 and 3 were conducted in 2024, and Experiment 2 was conducted in 2024 and 2025.

#### Participants

A sensitivity analysis conducted with G*Power 3.1 (Faul, Erdfelder, Lang, & Buchner, 2007) indicated that the smallest effect size that could be detected with 24 participants in a two-tailed paired-sample *t-*test with an alpha of 0.05 and 80% power was *d*_z_ = 0.60. This effect size was selected to match the methodological approach used in Huynh Cong & Kerzel (2022). The planned sample size was therefore set to 24 in the preregistration. Ethical approval for the study was granted by the Ethics Committee of the Faculty of Psychology and Educational Sciences of the University of Geneva (CUREG no. 20231004-331-2). All participants provided informed consent prior to the start of the experiment.

Thirty-two participants took part in the experiment to acquire class credits. Six participants were excluded because they did not meet the inclusion criteria specified in our preregistration (for more information on inclusion criteria, see the Procedure section). We decided to exclude one participant from all analyses (and not only from the model-fit analysis) due to an estimated guess rate of 0.78 in the 70% reliability invalid cue condition. Across all participants in that condition (*n* = 26), the mean guess rate was 0.085 (*SD* = 0.165). The next-highest value was 0.347. The remaining 25 participants (mean age ± *SD*, 22.0 ± 5.8 years; four men, 21 women) all reported normal or corrected-to-normal vision.

#### Apparatus

The stimuli were presented on a 22.5-inch in-plane switching (IPS) monitor (VIEWPixx Lite, 100 Hz, 1920 × 1200 pixels, standard backlight; VPixx Technologies Inc., Saint-Bruno, QC, Canada). Colors were measured with an i1Display Pro (VPixx Edition) colorimeter by X-Rite (Grand Rapids, MI); the xyY color coordinates are available in the OSF repository. Head position was stabilized with a chin/forehead rest at a viewing distance of 66 cm. Responses were collected using a standard universal serial bus (USB) wired mouse.

#### Stimuli

**Figure 1.**
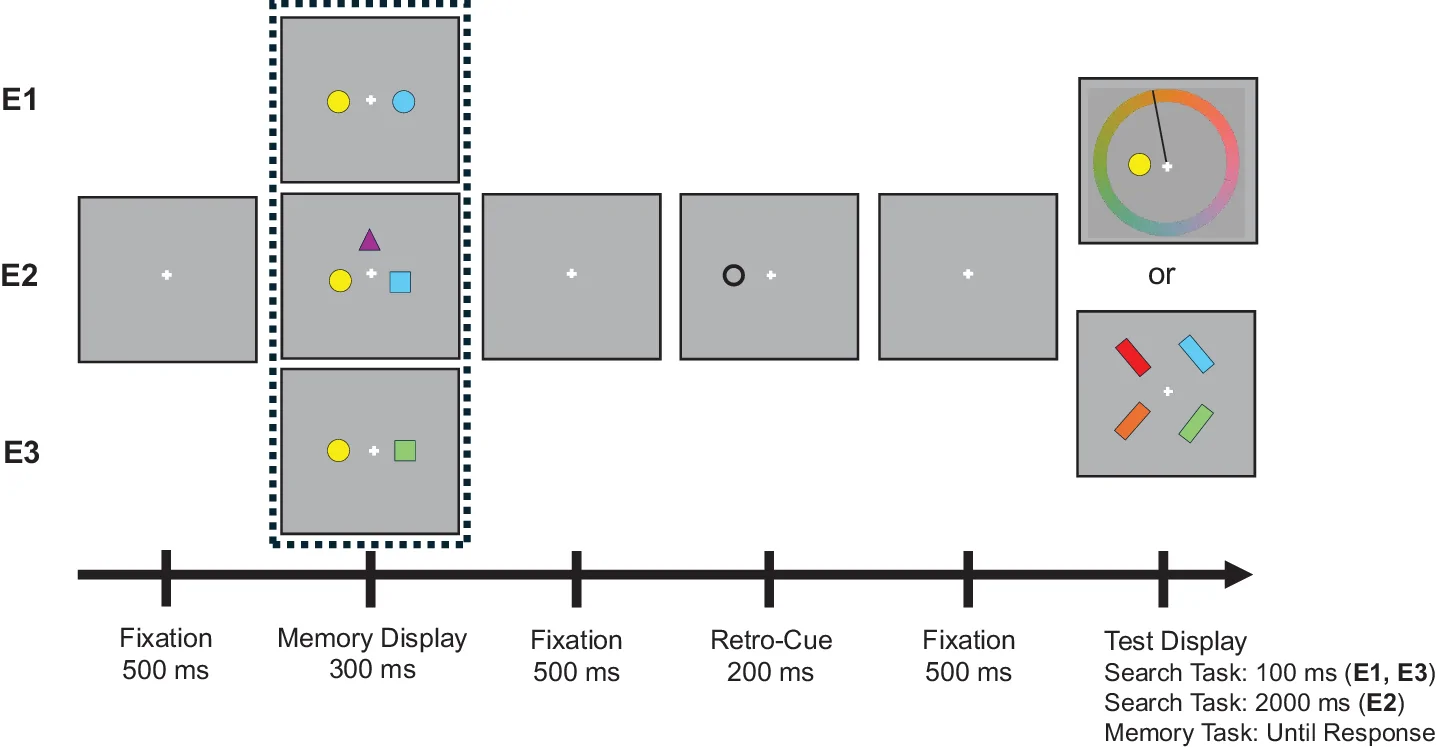
Experimental procedure. E1, E2, and E3, respectively, indicate Experiments 1, 2, and 3. Stimuli sizes and colors were adjusted for clarity. In all experiments, the retro-cue matched the position and shape of a memory item. On each trial, participants performed either a memory task (selecting the remembered color on a color wheel) or a search task (reporting the tilt of the bar matching the color of one item from the memory display). In Experiment 3, each target shape was associated with a task: The square was associated with the search task and the disk with the memory task.

Figure 1 illustrates the stimuli and shows the time course of a trial. Stimuli were presented on a light-gray background (15.88 cd/m^2^), and a white fixation cross measuring 0.18 degrees of visual angle (dva) was shown in the center of the display. We used a color wheel consisting of 360 colors drawn from the CIELAB color space (L* = 63, center a* = 9, center b* = 27, radius = 40) that were converted to RGB using the PsychoPy cielab2rgb function with the sRGB transfer function. On each trial, five colors were selected from the color wheel: One initial color was randomly sampled and, based on this color, four others each spaced 72° apart on the color wheel were selected. Each color was then jittered by up to ±6°, introducing slight variations while preserving similar color differences.

Each trial started with the fixation cross presented alone for 500 ms. The memory display then appeared for 300 ms. It consisted of two colored disks (0.37-dva radius; 0.43-dva^2^ area), each outlined in black with a 0.5-pixel outline and positioned 1.5 dva to the left and right of the fixation cross (center-to-center). The colors of these disks were randomly drawn (without replacement) from the five previously selected colors. Five hundred milliseconds after the offset of the memory display, the retro-cue appeared for 200 ms. It was a black unfilled disk (0.37-dva radius) shown at the location of one of the two disks from the memory display. The retro-cue was spatially valid when it indicated the item that was later probed and invalid when it indicated the other item. Retro-cue reliability referred to the probability that the cue was valid within a block: In 70%-reliability blocks, the cue was valid on 70% of trials, whereas, in 50%-reliability blocks, it was valid on half of the trials and was therefore uninformative. Another 500-ms interval separated the offset of the retro-cue and the onset of one of two possible response displays, corresponding either to a visual search task or to a memory recall task. The two tasks were equally likely and randomly intermixed across trials. The total interval from memory display offset to response display onset was 1200 ms.

On search-task trials, the response display appeared for 100 ms and consisted of four rectangular bars (1.30 × 0.33 dva; area, 0.43 dva^2^) displayed in the corners of a virtual square at 2 dva from fixation. The tilt of the bars was randomized, with two bars tilted 45° to the left and two tilted 45° to the right. The target bar was the one whose color matched that of one of the disks in the memory display. The three non-target bars were shown in colors that had not been used on that trial. Participants had to indicate the tilt of the target bar by clicking the left (for left-tilted) or right (for right-tilted) mouse button. After 15,000 ms, the response display timed out. When participants committed errors (anticipation, late response, or choice error), a feedback display appeared for 2000 ms (1000 ms in Experiment 2).

On memory-task trials, the response display consisted of an empty disk presented at one of the two positions from the memory display. Participants had to select the color previously shown at that location. The disk was surrounded by a color wheel with an inner radius of 4.5 dva and an outer radius of 5.5 dva. The color selected by mouse movement was highlighted by a black line between the central fixation mark and the color wheel. In addition, the outline disk was dynamically filled with the currently selected color. Participants confirmed their selection by pressing the left mouse button. On each trial, the orientation of the color wheel was randomly selected among multiples of 30° to avoid motor biases. This response display remained on screen until a response was made.

#### Procedure

Participants were invited to the laboratory for two sessions. During the first session, the task was explained, followed by a training phase consisting of four blocks of 40 trials each. Within each block, retro-cue reliability was held constant at either 50% or 70%. The reliability level assigned to the first two blocks was counterbalanced across participants and switched for the last two blocks. Participants were explicitly informed of the current retro-cue reliability at the beginning of each block, including whenever the reliability changed, and they were instructed to remember the two colors on each trial.

At the end of the training phase, performance was evaluated. As specified in the preregistration, participants could continue to the main experiment only if they achieved an accuracy above 80% in each condition of the search task and an average absolute angular deviation (hereafter, *recall error*) of less than 60° in each condition of the memory task. These criteria were applied to ensure adequate task engagement and understanding.

Participants who met these criteria then completed the main experiment across two sessions. The first part of the main experiment consisted of 320 trials, and the second session consisted of 640 additional trials, resulting in a total of 960 trials. Retro-cue reliability was manipulated between blocks. In the first session, reliability changed every 160 trials; in the second session, it changed every 320 trials. The order of reliability blocks was counterbalanced across participants. Throughout the experiment, the search and memory tasks were equally likely to occur. Across the full experiment, participants completed 480 trials in each task. In the 50%-reliability condition, each task included 120 valid and 120 invalid trials. In the 70%-reliability condition, each task included 168 valid and 72 invalid trials.

#### Analysis

We employed a 2 × 2 repeated-measures design. The two within-participant factors were retro-cue reliability (50%, 70%) and retro-cue validity (valid, invalid). The dependent variables were response times and accuracy in the search task and recall error in the memory task. The repeated-measures ANOVAs reported for Experiments 1, 2, and 3 used the same design. For post hoc analyses, all *p* values reported in the text were corrected using the Holm–Bonferroni method (Holm, 1979) to control for multiple comparisons. We also report Cohen's *d*_z_ (Cohen, 1988), with 95% confidence intervals (CIs) calculated using the standard parametric method based on the noncentral *t* distribution (Cumming, 2013). We had preregistered swap model analyses but ultimately decided to include them only in the Supplementary Materials for clarity. For each analysis, we applied specific exclusion criteria (described in each results section) to identify outliers. However, in line with Miller's (2023) suggestion that excluding outliers alters or skews results, we also include a table in the Supplementary Materials comparing statistical outcome with and without exclusion.

### Results

#### RTs in the search task

**Figure 2.**
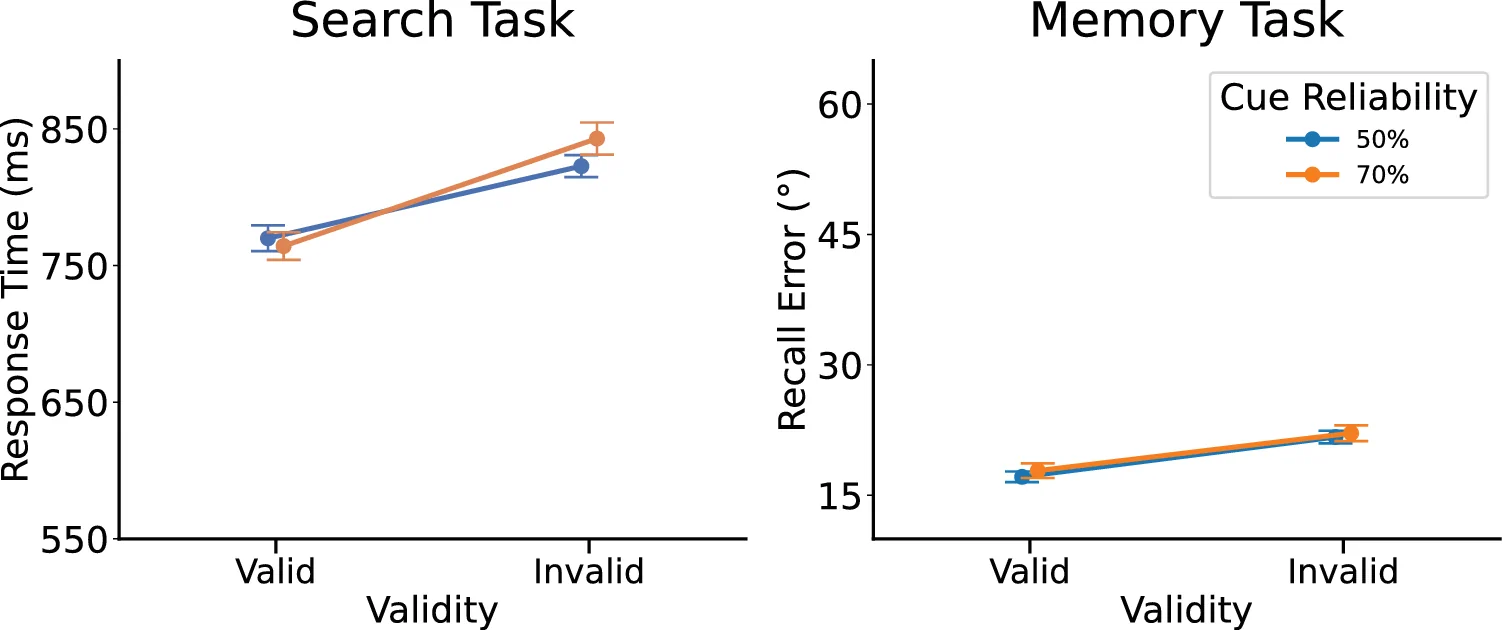
Results from Experiment 1. The left panel shows RTs (expressed in ms) as a function of cue reliability and validity. The right panel shows recall error (expressed in degrees) as a function of cue reliability and validity. Error bars represent 95% within-participant confidence intervals, calculated using Cousineau's (2005) method with Morey's (2008) correction.

Deviating from the preregistrations, we excluded trials with RTs shorter than 200 ms or longer than 2000 ms (3% of total trials) because very short RTs likely reflect anticipatory responses, and long RTs can indicate a loss of attention. However, including these data does not change the major outcomes. We also excluded trials with choice errors (8% of total trials). Next, as specified in the preregistration, we removed trials with RTs deviating more than 2.5 *SD* from the respective condition mean (3% of total trials). Although the preregistration mentioned we would analyze median response times, we instead chose to analyze averages. The median-based analyses were conducted but are not reported here; they are available on the OSF. All median-derived results yielded outcomes comparable to those of mean-derived results. Figure 2 shows average RTs.

The ANOVA revealed that the main effect of retro-cue validity, *F*(1, 24) = 25.61, *p* < 0.001, $\eta_{p}^{2}$ = 0.516, was modulated by an interaction between retro-cue validity and reliability, *F*(1, 24) = 7.30, *p* = 0.012, $\eta_{p}^{2}$ = 0.233, showing that the difference between valid and invalid cues was smaller with 50% than 70% cue validity (53 ms vs. 79 ms difference). Note that this interaction was not significant when removed outlier values and the participant removed due to his guess rate were kept. It should therefore be interpreted with great caution. To further investigate the interactions from the ANOVAs, we conducted post hoc paired-samples *t*-tests. These *t*-tests included comparisons between invalid and valid trials at each level of cue reliability, as well as comparisons between cue-reliability conditions separately for invalid and valid trials. All *p* values from post hoc comparisons were corrected for multiple testing using the Holm–Bonferroni method (Holm, 1979). This correction was applied separately for each experiment, across all post hoc comparisons relevant to the same dependent variable (e.g., RTs in the search task, recall error). Therefore, all of these *p* values were corrected for a family of two comparisons.

The difference between valid and invalid cues was significant at both levels of cue reliability. At 50% cue reliability, response times were slower following invalid cues than valid cues (823 ms vs. 770 ms), *t*(24) = 4.70, *p*_holm_ < 0.001, *d*_z_ = 0.94, 95% CI (0.44, 1.44). At 70% cue reliability, the same pattern was observed, with slower responses following invalid compared with valid cues (843 ms vs. 764 ms), *t*(24) = 4.91, *p*_holm_ < 0.001, *d*_z_ = 0.98, 95% CI (0.48, 1.49).

Additional contrasts showed that RTs did not differ significantly between the 50% and 70% reliability conditions either for invalid trials (823 vs. 843 ms), *t*(24) = −1.72, *p*_holm_ = 0.196, *d*_z_ = −0.34, 95% CI (−0.77, 0.08), or for valid trials (770 ms vs. 764 ms), *t*(24) = 0.52, *p*_holm_ = 0.606, *d*_z_ = 0.10, 95% CI (−0.31, 0.52). There was no main effect of retro-cue reliability, *F*(1, 24) = 0.50, *p* = 0.488, $\eta_{p}^{2}$ = 0.020.

#### Recall error in the memory task

For the analysis of the color wheel task, our dependent variable was the recall error. Deviating from the preregistrations, we only analyzed the average recall error for the sake of readability (see Figure 2). There was a main effect of validity, *F*(1, 24) = 17.51, *p* < 0.001, $\eta_{p}^{2}$ = 0.422, showing that recall errors were better following valid compared with invalid cues (17.48° vs. 21.93°). Contrary to our expectations, there was no interaction between retro-cue validity and reliability, *F*(1, 24) = 0.05, *p* = 0.826, $\eta_{p}^{2}$ = 0.002. The main effect of retro-cue reliability was also not significant, *F*(1, 24) = 0.94, *p* = 0.342, $\eta_{p}^{2}$ = 0.038.

### Exploratory analyses

#### Accuracy in the search task

We analyzed accuracy in the search task to ensure that potential differences in memory performance could not be attributed to speed-accuracy tradeoffs. Accuracy was lower with invalid than valid retro-cues (0.90 vs. 0.95), *F*(1, 24) = 10.18, *p* = 0.004, $\eta_{p}^{2}$ = 0.298. However, there was no main effect of retro-cue reliability, *F*(1, 24) = 0.34, *p* = 0.566, $\eta_{p}^{2}$ = 0.014, and no interaction between retro-cue reliability and validity, *F*(1, 24) = 0.60, *p* = 0.445, $\eta_{p}^{2}$ = 0.025.

#### RTs in the memory task

To explore potential factors influencing judgments in the color wheel task, we analyzed the mean RTs between the onset of the color wheel and the mouse click to confirm the selected color. We retained only values between 500 and 4000 ms (without applying a standard deviation-based exclusion criterion), which eliminated 6% of trials. We chose this interval because it represented the best compromise: It allowed us to retain the majority of trials while minimizing the influence of extreme outliers. The interaction between validity and retro-cue reliability, *F*(1, 24) = 5.14, *p* = 0.033, $\eta_{p}^{2}$ = 0.177, and the main effect of validity, *F*(1, 24) = 18.13, *p* < 0.001, $\eta_{p}^{2}$ = 0.430, were significant. We performed post hoc tests on the interaction levels, and the difference between invalid and valid cues was smaller with 50% reliability than with 70% reliability (50 ms vs. 112 ms difference). The difference between invalid and valid at 50% reliability approached significance (2135 vs. 2085 ms), *t*(24) = 1.94, *p*_holm_ = 0.065, *d*_z_ = 0.37, 95% CI (−0.02, 0.77), but was significant at 70% reliability (2152 ms vs. 2040 ms), *t*(24) = 5.43, *p*_holm_ < 0.001, *d*_z_ = 1.05, 95% CI (0.57, 1.53). The main effect of retro-cue reliability was not significant, *F*(1, 24) = 0.11, *p* = 0.746, $\eta_{p}^{2}$ = 0.004.

### Discussion

In Experiment 1, we investigated whether the reliability of retro-cues modulates performance in visual search and memory in a graded fashion. In the search task, we observed a validity effect that appeared to be modulated by cue reliability: The benefit of a valid cue was larger when reliability was high (70%) compared with when it was uninformative (50%). Rather than acting as a binary switch, the results suggest that the attentional system adjusts the distribution of priority based on the predictive utility of the cue. However, the interaction was sensitive to the exclusion criteria and is therefore not robust; therefore, Experiment 1 offers only limited evidence for an effect of cue reliability on search performance.

In the memory task, however, the pattern was different. Although we found a clear benefit of valid cues on recall error, this effect did not interact with reliability. At first glance, this absence of interaction could suggest a dissociation between memory and guidance, but opposite to previous findings (Dube & Al-Aidroos, 2019). Perhaps priority tuning refines the guiding template (search) without equally sharpening the memory representation. However, an alternative possibility is that the memory task with only two items was too easy, leading to a ceiling effect that masked subtle shifts in precision. Consistent with this hypothesis, the exploratory analysis of memory response times showed the predicted interaction, with a larger validity benefit at 70% reliability. This suggests that the “tuning” mechanism may have been engaged during the memory task, manifesting in processing speed rather than precision due to the low load. To adjudicate between a true dissociation and a ceiling effect, it was necessary to increase task difficulty.

## Experiment 2

Experiment 2 was designed to examine whether the effects of retro-cue reliability on prioritization depend on the level of competition in VWM. Previous studies on cue reliability and VWM performance have often used low set sizes, typically two items (Dube & Al-Aidroos, 2019; Gunseli et al., 2015). Although such designs maximize baseline performance, they also limit competition between representations, making it difficult to assess whether reliability-based prioritization operates when memory resources are more strongly constrained.

To address this limitation, we increased the memory load to three items. Increasing set size strengthens competition between stored representations and therefore provides a more sensitive test of whether cue reliability modulates prioritization under higher interference. With three items, an uninformative retro-cue corresponds to chance-level validity (33%), whereas the informative condition remains fixed at 70%. To facilitate the maintenance of three items and reduce perceptual ambiguity, colors were presented on distinct shapes (disk, square, and triangle).

The key question was whether increasing competition would lead to a reliability-dependent modulation of performance in the memory task that was not observed under low load in Experiment 1. If prioritization depends on cue reliability in a graded manner, the validity effect should be larger in the 70% condition than in the 33% condition for both the search task and the memory task. Observing such an interaction in memory performance would argue against a strict dissociation between storage and guidance and support the idea that probabilistic information influences both functions under conditions of sufficient competition.

### Methods

#### Participants

A total of 56 participants were recruited in exchange for course credit. Of these, 30 participants were excluded because they did not meet the accuracy criterion during the training phase. This exclusion rate was high despite lowering the search-task accuracy requirement from 80% to 70% to accommodate the increased difficulty associated with the higher memory load. In addition, one participant did not complete the second session, leaving a final sample of 25 participants for analysis (mean age ± SD, 21.2 ± 4.6 years; three men, 22 women).

#### Stimuli and procedure

The stimuli and procedure were identical to those of Experiment 1, except for the following changes. In the memory display, a disk (radius: 0.37 dva), a square (side length of 0.66 dva), and an equilateral triangle (side length of 1.00 dva) were presented at an eccentricity of 2.50 dva, arranged at three equally spaced locations (120° apart). The spatial locations were fixed across trials, but the shape assigned to each location varied randomly.

To accommodate the higher task difficulty of maintaining three items and to prevent floor-effect performance, the search display duration was increased from 100 ms to 2000 ms. This adjustment was motivated by informal pilot testing, which revealed that the 100-ms display used in Experiment 1 reduced performance to chance levels under the higher memory load. We also used six colors instead of five, resulting in a minimum angular separation of 60°, with each color jittered by ±6°. Retro-cue reliability was manipulated across blocks (33% vs. 70%). Accordingly, out of the 480 trials per task, there were 80 valid and 160 invalid trials in the 33% reliability condition, and 168 valid and 72 invalid trials in the 70% reliability condition. Finally, the retro-cue matched the shape presented at the cued location. Each shape and each location were cued and probed equally often (one third of the trials).

### Results

#### RTs in the search task

**Figure 3.**
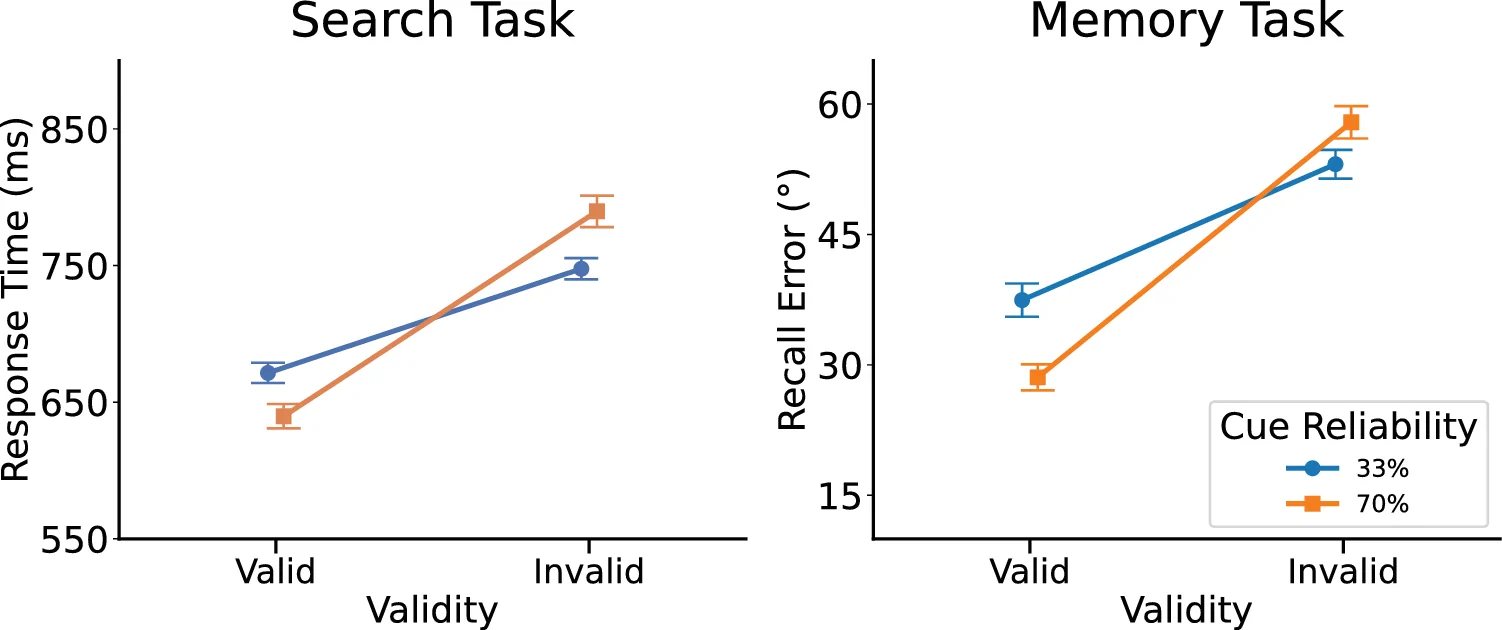
Results from Experiment 2. The left panel shows RTs (expressed in ms) as a function of cue reliability and validity. The right panel shows recall error (expressed in degrees) as a function of cue reliability and validity. Error bars represent 95% within-participant confidence intervals, calculated using Cousineau's (2005) method with Morey's (2008) correction.

RT preprocessing followed the same criteria as in Experiment 1. Briefly, trials with anticipatory or excessively slow responses, choice errors, and outlier RTs (>2.5 *SD* from the condition mean) were excluded. Figure 3 shows average RTs. There was a main effect of retro-cue validity, *F*(1, 24) = 85.04, *p* < 0.001, $\eta_{p}^{2}$ = 0.780, as well as an interaction effect between retro-cue validity and reliability, *F*(1, 24) = 27.25, *p* < 0.001, $\eta_{p}^{2}$ = 0.532. The interaction indicates that the difference between valid and invalid cues was smaller with 33% reliability than with 70% reliability (76 ms vs. 150 ms difference). The difference between valid (671 ms) and invalid (748 ms) trials was significant at 33% reliability, *t*(24) = −6.73, *p*_holm_ < 0.001, *d*_z_ = −1.35, 95% CI (−1.92, −0.77), and at 70% reliability (640 ms vs. 790 ms), *t*(24) = −9.08, *p*_holm_ < 0.001, *d*_z_ = −1.82, 95% CI (−2.50, −1.14).

Additional contrasts showed that RTs differed significantly between the 33% and 70% reliability conditions for both invalid and valid trials. For invalid trials, responses were slower in the 70% reliability condition (790 ms) than in the 33% reliability condition (748 ms), *t*(24) = 3.56, *p*_holm_ = 0.002, *d*_z_ = 0.71, 95% CI (0.25, 1.18). Conversely, for valid trials, responses were faster in the 70% reliability condition (640 ms) than in the 33% reliability condition (671 ms), *t*(24) = −4.44, *p*_holm_ < 0.001, *d*_z_ = −0.89, 95% CI (−1.38, −0.40). There was no main effect of cue reliability, *F*(1, 24) = 0.59, *p* = 0.450, $\eta_{p}^{2}$ = 0.024.

#### Recall error in the memory task

The average recall errors are also shown in Figure 3. There was a main effect of retro-cue validity, *F*(1, 24) = 77.36, *p* < 0.001, $\eta_{p}^{2}$ = 0.763, as well as an interaction between retro-cue validity and reliability, *F*(1, 24) = 38.93, *p* < 0.001, $\eta_{p}^{2}$ = 0.619. The interaction indicates that the difference between valid and invalid cues was smaller at 33% reliability than at 70% reliability (16° vs. 29° difference). The difference between valid (37°) and invalid (53°) trials was significant at 33% reliability, *t*(24) = −5.44, *p*_holm_ < 0.001, *d*_z_ = −1.09, 95% CI (−1.61, −0.56), and at 70% reliability (29° vs. 58°), *t*(24) = −10.92, *p*_holm_ < 0.001, *d*_z_ = −2.18, 95% CI (−2.95, −1.41).

Additional contrasts showed that recall error differed significantly between the 33% and 70% reliability conditions both for invalid and valid trials. For invalid trials, recall error was larger in the 70% reliability condition (58°) than in the 33% reliability condition (53°), *t*(24) = 2.92, *p*_holm_ = 0.007, *d*_z_ = 0.58, 95% CI (0.14, 1.03). Conversely, for valid trials, recall error was lower in the 70% reliability condition (29°) than in the 33% reliability condition (37°), *t*(24) = −5.86, *p*_holm_ < 0.001, *d*_z_ = −1.17, 95% CI (−1.71, −0.63). The main effect of retro-cue reliability was not significant, *F*(1, 24) = 3.17, *p* = 0.088, $\eta_{p}^{2}$ = 0.117.

### Exploratory analyses

#### Accuracy in the search task

Similar to Experiment 1, we analyzed accuracy in the search task. There was a main effect of retro-cue validity, *F*(1, 24) = 102.91, *p* < 0.001, $\eta_{p}^{2}$ = 0.811, which was modulated by an interaction between retro-cue validity and reliability, *F*(1, 24) = 20.06, *p* < 0.001, $\eta_{p}^{2}$ = 0.455. The interaction showed that the difference between valid and invalid cues was smaller with 33% reliability than with 70% reliability (0.08 vs. 0.14 difference). The difference between valid (0.82) and invalid (0.74) trials was significant with 33% reliability, *t*(24) = 9.89, *p*_holm_ < 0.001, *d*_z_ = 1.98, 95% CI (1.30, 2.65), and with 70% reliability (0.82 vs. 0.68), *t*(24) = 5.47, *p*_holm_ < 0.001, *d*_z_ = 1.09, 95% CI (0.60, 1.59). There was no main effect of retro-cue reliability, *F*(1, 24) = 1.06, *p* = 0.313, $\eta_{p}^{2}$ = 0.042.

#### RTs in the memory task

As in Experiment 1, we analyzed RTs to explore whether retro-cue validity and reliability influenced response speed during the memory task. There was a main effect of retro-cue validity, *F*(1, 24) = 141.78, *p* < 0.001, $\eta_{p}^{2}$ = 0.855. This effect was modulated by an interaction between retro-cue validity and reliability, *F*(1, 24) = 32.66, *p* < 0.001, $\eta_{p}^{2}$ = 0.576, indicating that the validity benefit was smaller at 33% reliability than at 70% reliability (159 ms vs. 327 ms). The difference between valid (1613 ms) and invalid (1454 ms) trials was significant at 33% reliability, *t*(24) = 6.80, *p*_holm_ < 0.001, *d*_z_ = 1.36, 95% CI (0.82, 1.90), and at 70% reliability (1747 ms vs. 1420 ms), *t*(24) = 11.98, *p*_holm_ < 0.001, *d*_z_ = 2.40, 95% CI (1.63, 3.17). The main effect of retro-cue reliability was not significant, *F*(1, 24) = 2.99, *p* = 0.097, $\eta_{p}^{2}$ = 0.111.

### Discussion

Experiment 2 was designed to follow up on Experiment 1, where no interaction between retro-cue validity and reliability was observed in recall error under low memory load. One possible interpretation of this pattern is that, with only two items, participants could maintain both representations with sufficient fidelity, such that differences in prioritization as a function of cue reliability were not expressed in recall performance. By increasing the memory load to three items, we increased competition between stored representations. Under these conditions, we observed a clear interaction between retro-cue validity and reliability in both tasks.

In the search task, responses were faster following valid than invalid retro-cues, and this validity effect was larger when cue reliability was high. At 70% reliability, valid cues led to substantially greater response time benefits than at 33% reliability. This pattern indicates that cue reliability modulated the extent to which a representation influenced attentional guidance, rather than acting as a simple all-or-none switch.

A similar interaction was observed in the memory task. Recall performance differed more strongly between valid and invalid trials when retro-cues were reliable than when they were uninformative. Importantly, this larger validity effect reflected both improved performance on valid trials and reduced performance on invalid trials under high reliability, consistent with a redistribution of priority across representations rather than a uniform gain. Together, these results suggest that the relationship between memory-based guidance and memory recall is sensitive to task demands and competitive context. When competition between items is sufficiently strong, cue reliability modulates the magnitude of validity effects in both attentional guidance and memory performance.

However, one limitation when comparing Experiments 1 and 2 is that the duration of the search display was increased from 100 ms to 2000 ms in Experiment 2 to accommodate the greater task difficulty. Consequently, differences in search performance between the two experiments cannot be attributed exclusively to the increase in memory load.

In addition to memory load, task structure may also influence the expression of reliability effects. In Experiments 1 and 2, participants did not know during the retention interval whether a given item would be tested in a search task or a recall task. This uncertainty may have encouraged a more general maintenance strategy that balanced the requirements of both tasks. Experiment 3 was designed to test this possibility by removing the shape-task uncertainty by using fixed associations.

## Experiment 3

In Experiments 1 and 2, participants encoded multiple items without knowing which of them would later be relevant for visual search or for memory recall. Although the task itself (search vs. memory) was equally likely on each trial, the functional role of each memorized item was not specified during encoding and maintenance.

According to Guided Search 6.0 (Wolfe, 2021), visual search relies on a distinction between a guiding template and a target template. The guiding template biases attention toward objects with task-relevant features, whereas the target template provides a more precise representation used to determine whether a selected object is in fact the target. Wolfe (2021) further suggests that the guiding template may plausibly reside in VWM, particularly when guidance is based on simple features such as color.

Experiment 3 was designed to apply this distinction more directly to our task. By assigning one shape to the search task and the other to the memory task, each memorized item had a stable role during maintenance. The search-associated item was more likely to support attentional guidance, whereas the memory-associated item was more likely to be maintained for later recall. This manipulation did not reduce uncertainty about which task would occur on a given trial, but it did reduce uncertainty about the role of each item. It therefore allowed us to test whether cue reliability would have clearer effects when participants could prepare the two representations for different functions in advance. Such a pattern would be consistent with Wolfe's distinction between guidance and target identification. According to flexible prioritization accounts (e.g., Gunseli et al., 2015; Huynh Cong & Kerzel, 2021), processing resources can be distributed across representations as a function of their expected relevance. By manipulating retro-cue reliability in this context, we tested whether reliability-based prioritization operates similarly when representations have distinct, predefined functional roles. If probabilistic prioritization is a general mechanism, cue reliability should modulate performance in both tasks.

### Methods

#### Participants

Thirty participants took part in the experiment to acquire class credits. Four participants were excluded because they did not reach the performance criteria in the training session (see Experiment 1). In addition, two participants did not come back for the second part. The remaining 24 participants (mean age ± SD, 22.6 ± 9.2 years; three men, 21 women) all reported normal or corrected-to-normal vision.

#### Stimuli and procedure

The stimuli and procedure were largely similar to Experiment 1 with the following modifications. In the memory display, instead of presenting two colored disks, we presented one disk and one square. Crucially, the square was associated with the search task and the disk with the memory task. That is, the color of the target in the search task always matched that of the square, and the to-be-remembered color in the memory task was always that of the disk. Moreover, because there were only two targets, the cue reliabilities were similar to those of Experiment 1 (50% and 70%).

### Results

#### RTs in search task

**Figure 4.**
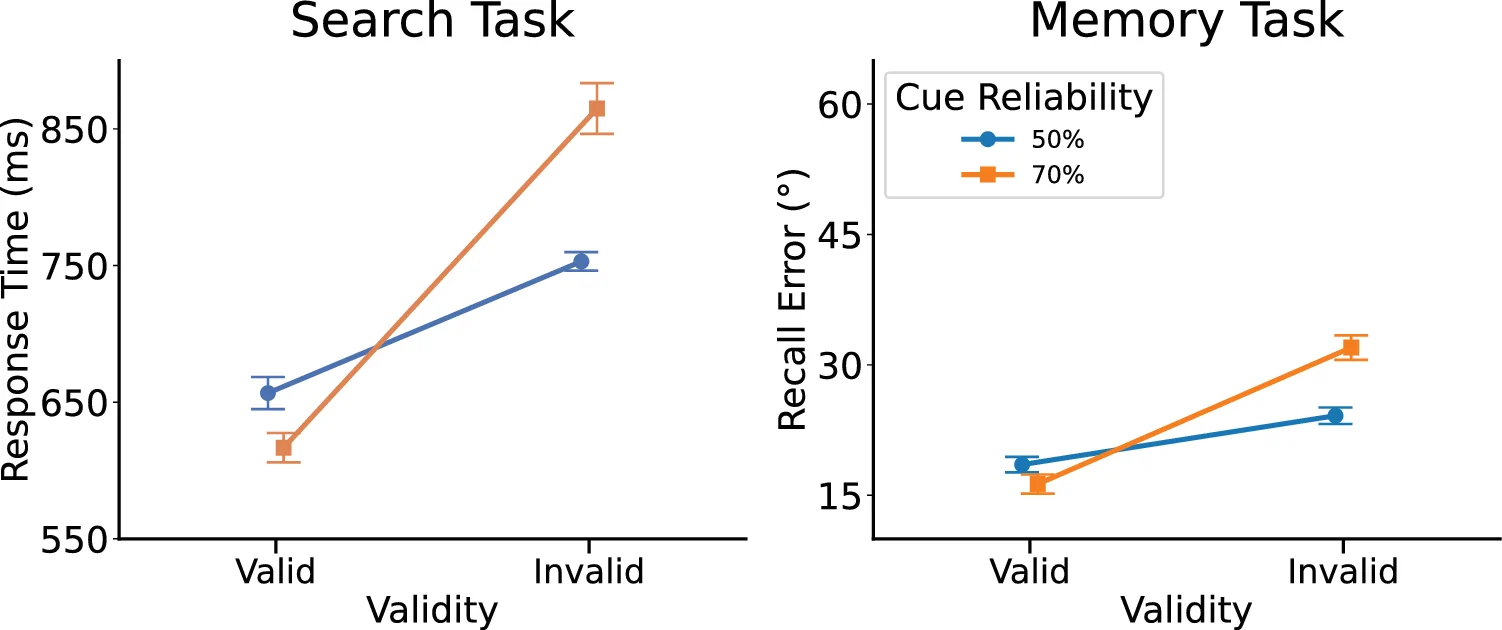
Results from Experiment 3. The left panel shows RTs (expressed in ms) as a function of cue reliability and validity. The right panel shows recall error (expressed in degrees) as a function of cue reliability and validity. Error bars represent 95% within-participant confidence intervals, calculated using Cousineau's (2005) method with Morey's (2008) correction.

Trials with anticipations or late responses (2% of trials), response errors (9%), and outlier RTs (3%) were removed. Figure 4 shows average RTs. There was a main effect of retro-cue validity, *F*(1, 23) = 110.41, *p* < 0.001, $\eta_{p}^{2}$ = 0.828. This effect was modulated by an interaction between retro-cue validity and reliability, *F*(1, 23) = 65.37, *p* < 0.001, $\eta_{p}^{2}$ = 0.740, indicating that the validity benefit was smaller at 50% reliability than at 70% reliability (96 ms vs. 248 ms). The difference between valid and invalid trials was significant at 50% reliability (657 ms vs. 753 ms), *t*(23) = −7.85, *p*_holm_ < 0.001, *d*_z_ = −1.60, 95% CI (−2.25, −0.96), and at 70% reliability (617 ms vs. 865 ms), *t*(23) = –10.46, *p*_holm_ < 0.001, *d*_z_ = −2.13, 95% CI (−2.91, −1.36).

Additional contrasts showed that RTs differed significantly, between the 50% and 70% reliability conditions for both invalid and valid trials. For invalid trials, responses were slower in the 70% reliability condition (865 ms) than in the 50% reliability condition (753 ms), *t*(23) = 6.29, *p*_holm_ < 0.001, *d*_z_ = 1.28, 95% CI (0.71, 1.86). Conversely, for valid trials, responses were slower in the 50% reliability condition (657 ms) than in the 70% reliability condition (617 ms), *t*(23) = 3.77, *p*_holm_ = 0.001, *d*_z_ = 0.77, 95% CI (0.29, 1.25). There was also a main effect of retro-cue reliability, *F*(1, 23) = 10.24, *p* = 0.004, $\eta_{p}^{2}$ = 0.308; however, this effect was not significant when excluded values were retained.

#### Recall error in the memory task

Figure 4 shows average recall error. There was a main effect of retro-cue validity, *F*(1, 23) = 42.45, *p* < 0.001, $\eta_{p}^{2}$ = 0.649. This effect was modulated by an interaction between retro-cue validity and reliability, *F*(1, 23) = 56.18, *p* < 0.001, $\eta_{p}^{2}$ = 0.710, indicating that the difference between valid and invalid cues was smaller at 50% reliability than at 70% reliability (6° vs. 16°). The difference between valid and invalid trials was significant at 50% reliability (19° vs. 24°), *t*(23) = −3.99, *p*_holm_ = 0.001, *d*_z_ = −0.81, 95% CI (−1.30, −0.32), and at 70% reliability (16° vs. 32°), *t*(23) = −7.60, *p*_holm_ < 0.001, *d*_z_ = −1.55, 95% CI (−2.19, −0.92).

Additional contrasts showed that recall error differed significantly between the 50% and 70% reliability conditions for both invalid and valid trials. For invalid trials, recall error was larger in the 70% reliability conditions (32°) than in the 50% reliability conditions (24°), *t*(23) = 6.05, *p*_holm_ < 0.001, *d*_z_ = 1.23, 95% CI (0.67, 1.80). Conversely, for valid trials, recall error was larger in the 50% reliability condition (19°) than in the 70% reliability condition (16°), *t*(23) = 3.88, *p*_holm_ = 0.001, *d*_z_ = 0.79, 95% CI (0.31, 1.28). There was also a main effect of retro-cue reliability, *F*(1, 23) = 14.12, *p* = 0.001, $\eta_{p}^{2}$ = 0.380.

### Exploratory analyses

#### Accuracy in the search task

As in previous experiments, we analyzed accuracy in the search. There was a main effect of retro-cue validity, *F*(1, 23) = 47.27, *p* < 0.001, $\eta_{p}^{2}$ = 0.673. This effect was modulated by an interaction between retro-cue validity and reliability, *F*(1, 23) = 27.10, *p* < 0.001, $\eta_{p}^{2}$ = 0.541, indicating that the difference between valid and invalid cues was smaller at 50% reliability than at 70% reliability (0.07 vs. 0.13). The difference between valid and invalid trials was significant at 50% reliability (0.95 vs. 0.88), *t*(23) = 5.38, *p*_holm_ < 0.001, *d*_z_ = 1.10, 95% CI (0.59, 1.61), and at 70% reliability (0.96 vs. 0.83), *t*(23) = 7.35, *p*_holm_ < 0.001, *d*_z_ = 1.50, 95% CI (0.92, 2.08). There was also a main effect of retro-cue reliability, *F*(1, 23) = 7.74, *p* = 0.011, $\eta_{p}^{2}$ = 0.252.

#### RTs in the memory task

We analyzed RTs in the color wheel task to explore whether retro-cue reliability and validity influenced the speed of memory-based responses. As before, we excluded trials with RTs below 500 and above 4000 ms (5% of trials). RTs were shorter with valid than invalid retro-cues (1844 ms vs. 2008 ms), *F*(1, 23) = 58.76, *p* < 0.001, $\eta_{p}^{2}$ = 0.719. The main effect of cue reliability, *F*(1, 23) = 0.57, *p* = 0.459, $\eta_{p}^{2}$ = 0.024, was not significant, but the interaction was, *F*(1, 23) = 44.02, *p* < 0.001, $\eta_{p}^{2}$ = 0.657. The difference between valid and invalid cues was smaller with 50% (difference of 71 ms; 1921 ms vs. 1992 ms), *t*(23) = −4.09, *p*_holm_ < 0.001, *d*_z_ = −0.84, 95% CI (−1.30, −0.37), than with 70% retro-cue validity (difference of 246 ms; 1792 ms vs. 2038 ms), *t*(23) = −8.43, *p*_holm_ < 0.001, *d*_z_ = −1.72, 95% CI (−2.35, −1.09).

### Discussion

In Experiment 3, we examined how probabilistic prioritization operates when memorized items are associated with distinct functional roles. Unlike Experiments 1 and 2, each shape was consistently linked to a specific task (search or memory), but the task itself remained unpredictable on each trial. This manipulation allowed us to test whether reliability-based prioritization depends on knowing the functional role of a representation, independently of knowing which task will be required.

In the search task, we again observed a validity effect that interacted with cue reliability. The difference between valid and invalid trials was larger in the 70% reliability condition than in the 50% condition. This pattern indicates that cue reliability modulated the extent to which the cued item guided visual search, even when participants did not know in advance whether the search task would occur.

A similar interaction was observed in the memory task. Recall errors differed more strongly between valid and invalid trials in the 70% reliability condition than in the 50% condition. However, the absence of a corresponding interaction in Experiment 1 should not be interpreted as evidence that the effect was absent under that task structure. It may instead reflect a type II error or ordinary experiment-to-experiment variability.

Experiment 3 provides an additional demonstration that reliability effects can be observed when memorized items have predefined functional roles. However, Experiments 1 and 3 were not designed as a direct between-experiment manipulation of shape–task association alone. Therefore, although the stronger reliability effects observed in Experiment 3 are consistent with the possibility that predefined task roles helped participants use memorized information in a task-specific way, they should not be interpreted as direct evidence that shape–task associations caused these stronger effects.. The comparison with Experiment 1 should therefore be considered descriptive. This pattern is also compatible with the Guided Search 6.0 (Wolfe, 2021) distinction between guiding and target templates. When task roles were stably assigned, participants may have been better able to maintain one representation for attentional guidance and the other for recall. However, the present design did not directly test this distinction.

Cue reliability influenced performance in both tasks, consistent with the idea that priority allocation is sensitive to expected utility, but without requiring advance knowledge of the upcoming task. These findings are consistent with a view in which prioritization is flexible and context dependent, rather than being tied to a rigid distinction between representational states or to advance certainty about the upcoming task

## General discussion

Across three experiments, we examined whether attentional priority in VWM operates in an all-or-none manner or varies as a function of cue reliability. Overall, retro-cue validity influenced both visual search and memory recall. In several conditions, the effect of validity increased with cue reliability. However, this pattern did not emerge uniformly across all experiments, and its expression depended on task structure and memory load. Thus, rather than concluding that processing capacity directly tracks cue reliability across tasks, our findings indicate that reliability can modulate performance under specific conditions.

The pattern across experiments highlights the importance of task demands. In Experiment 1 (two items), we observed an interaction between cue validity and reliability in search RTs, but not in recall error. This absence of interaction in recall does not necessarily suggest that prioritization was present but hidden. An alternative possibility is that, when the task is relatively easy, allocating additional resources based on reliability may provide little benefit, and participants may therefore not engage in reliability-dependent tuning of memory precision. Under low load, maintaining two items may not require strong redistribution of resources.

Experiment 2 increased memory load to three items, thereby increasing competition between stored representations. Under these conditions, cue validity interacted with reliability in both search performance and recall error. Importantly, this result does not demonstrate that the effect observed in Experiment 2 was already present but undetected in Experiment 1. Rather, it suggests that reliability-dependent modulation becomes more apparent when memory demands are higher and competition is stronger. In other words, the increased load created conditions under which differential allocation based on cue reliability had measurable behavioral consequences.

Experiment 3 addressed a different factor: the functional role of memorized items. By introducing stable shape–task associations, each item was consistently linked to either search guidance or memory recall. Reliability effects were observed in both tasks under this structure. However, this does not suggest that prioritization is uniquely sensitive to task uncertainty, as reliability effects were also present in Experiment 2. Instead, Experiment 3 shows that reliability-dependent modulation can also be observed at low load when representations have predefined functional roles. However, the apparent difference between Experiments 1 and 3 cannot establish that predefined task roles enhanced reliability-dependent prioritization. The absence of an interaction in recall error in Experiment 1 may reflect a type II error, and the difference between experiments may also reflect ordinary experiment-to-experiment variability. The design does not allow a formal test of task uncertainty per se, but the results are compatible with the possibility that task structure influences how probabilistic information is used. This interpretation is also consistent with Guided Search 6.0 (Wolfe, 2021), which distinguishes between a guiding template and a target template. When task roles were fixed across trials, participants may have been better able to maintain one item in a format suited to guiding search and the other in a format suited to later recall. Although the present data do not directly test this theoretical distinction, they show that reliability-dependent effects can be observed when memorized items have predefined task roles. Whether these predefined roles contributed to the magnitude of the effects cannot be determined from the present comparison.

One aim of this study was to clarify the relationship between memory precision (target template) and attentional guidance (guiding template), an issue that has produced mixed findings in previous work. Previous studies have shown that probabilistic retro-cues can improve memory performance without producing corresponding effects on attentional capture, particularly when cues are not fully reliable (e.g., Dube et al., 2019). Similarly, Dube and Al-Aidroos (2019) argued that prioritization for recall and prioritization for search are dissociable. Our findings do not resolve this debate, but they indicate that, under certain conditions (specifically, higher load or structured task roles), reliability modulates both recall and search performance. This suggests that the apparent dissociation reported previously may depend on task context and measurement sensitivity.

Importantly, the graded pattern observed here should be interpreted cautiously. Each experiment manipulated only two levels of reliability, which limits conclusions about whether prioritization varies continuously or categorically. Our data show that higher reliability was associated with larger validity effects in several conditions, but they do not establish the precise functional form of this relationship. Testing multiple intermediate reliability levels would be necessary to determine whether the effect scales linearly or follows another function.

The present findings are compatible with accounts proposing flexible allocation of WM resources (Bahle, Thayer, Mordkoff, & Hollingworth, 2020; Huynh Cong & Kerzel, 2021). Rather than assuming that items exist exclusively in discrete active versus accessory states, these frameworks suggest that representations can differ in the amount of processing gain they receive. Our results are consistent with this idea in that higher cue reliability was often associated with larger differences between valid and invalid trials. However, because reliability was manipulated dichotomously within each experiment, the data do not directly adjudicate between continuous and state-based models. Instead, they indicate that binary state accounts may not fully capture the range of observed behavior under probabilistic cueing.

We also considered interpretations inspired by probabilistic or optimal-observer frameworks, which propose that cognitive systems incorporate expectations into resource allocation (e.g., Chetverikov & Kristjansson, 2022; Ma, Husain, & Bay, 2014). From this perspective, retro-cues provide information about the likelihood that a representation will be relevant. Our findings are consistent with the idea that such probabilistic information influences performance. However, we did not directly test computational models of Bayesian updating, and our data should therefore be understood as behaviorally consistent with, rather than as confirming, these theoretical accounts.

Several limitations warrant consideration. First, reliability-dependent effects observed at the group level cannot uniquely distinguish between a continuous change in the strength of prioritization and a probabilistic all-or-none strategy. For example, participants may have prioritized the cued item only on a proportion of trials corresponding approximately to cue reliability. Such probability matching would produce an aggregate validity effect that resembles a graded prioritization benefit. Thus, our results show that cue reliability modulated performance, but they do not by themselves establish if this modulation reflects continuous trial-by-trial tuning or probabilistic switching between discrete states.

Second, only two reliability levels were tested per experiment (e.g., 33% vs. 70%). This limits our ability to characterize the functional relationship between probability and prioritization. Also, conclusions about the role of task structure are based on comparisons across experiments rather than formal within-experiment manipulations. A future design directly manipulating task certainty within participants would provide a stronger test. Third, the high exclusion rate in Experiment 2 raises concerns about generalizability. The final sample may over-represent participants with stronger cognitive control or higher motivation, and reliability-dependent modulation may differ in broader populations.

Finally, Experiment 2 differed from Experiment 1 not only in memory load, but also in search-display duration. The longer display duration in Experiment 2 may have allowed additional processing of the search array and could have increased the sensitivity of the search measure to retro-cue effects, especially because retro-cue–related updating processes unfold over time (Schneider, Mertes, & Wascher, 2016). Therefore, the stronger reliability effects in Experiment 2 may not be attributed uniquely to increased memory load.

In sum, the present study indicates that retro-cue reliability can modulate both visual search and memory recall under specific task conditions. Rather than demonstrating a strict dissociation or a definitive coupling between precision and guidance, our results suggest that their relationship depends on memory load and task structure. These findings contribute to ongoing discussions about how WM resources are allocated under uncertainty, and they highlight the importance of considering contextual factors when interpreting reliability-based prioritization effects.

### Constraints on generality

The participants in our study were undergraduate psychology students from the University of Geneva, all of whom were adults, with a majority being young women. As such, the generalizability of our findings to broader age groups, educational backgrounds, or cultural contexts remains uncertain. Moreover, our experimental design employed a specific form of retro-cueing based on color–location associations and manipulated cue reliability within a limited range (33%–70%). Different cueing modalities, feature dimensions, or levels of cue reliability might yield different results. Thus, caution is warranted when extending these conclusions to other populations, task formats, or attentional cueing paradigms.

## Supplementary Material

Supplement 1
