# Supplementary material for: Probabilistic tuning of attentional templates: Evidence for flexible prioritization in visual working memory
Source: J Vis. 2026 Aug 21;26(8):6. doi: 10.1167/jov.26.8.6 (PMC13505800; doi:10.1167/jov.26.8.6)
Supplement: Supplementary file 1 [file jovi-26-8-6_s001.pdf]

Supplementary for *Probabilistic Tuning of Attentional Templates: Evidence for Flexible Prioritization in Visual Working Memory*

Experiment 1

Swap model. We submitted the memory task's results to Bays et al. (2009) mixture-swap model in order to disentangle the sources of error. Note that we used Bays et al. (2009) mixture-swap model, but that other swap models exist (Bays, 2016; see Williams et al., 2022, 2023 for a comparison of these models). p_{guess} reflects the proportion of random guesses, *SD* reflects the precision of responses directed toward the correct target and p_{swap} reflects the proportion of responses corresponding to the non-probed item. We applied the swap model to move beyond a simple analysis of overall error magnitude and to dissociate the potential underlying sources of error. This decomposition was critical to determine whether our experimental manipulations specifically affected memory precision, the likelihood of item confusion, or the propensity to guess randomly. In particular, an effect on *SD* indicates a modulation of the quality of memory representations, consistent with flexible resource allocation. An effect on p_{swap}, on the other hand, reflects changes in the probability of selecting the correct item, suggesting differences in selection or binding. These parameters were fit with the MemToolbox (Suchow et al., 2013).

For guess rates (p_{guess}), there was no main effect of the cue reliability, $F(1, 24) = 0.09$, $p = .762$, $\eta_p^2 = .004$, nor was there a main effect of validity, $F(1, 24) = 1.94$, $p = .177$, $\eta_p^2 = .075$. The interaction between the cue reliability and the validity was also not significant, $F(1, 24) = 0.005$, $p = .944$, $\eta_p^2 < .001$. Mean guess rate was .048.

For swap rates (p_{swap}), there was no main effect of the retro-cue reliability, $F(1, 24) = 1.56$, $p = .224$, $\eta_p^2 = .061$, but swap rates were higher with invalid than with valid retro-cues

(.045 vs .013), $F(1, 24) = 5.70$, $p = .025$, $\eta_p^2 = .192$. The interaction between cue reliability and validity was not significant, $F(1, 24) = 0.15$, $p = .706$, $\eta_p^2 = .006$. Mean swap rate was .029.

For the *SD*, there was no main effect of cue reliability, $F(1, 24) = 0.73$, $p = .400$, $\eta_p^2 = .030$, but *SD*s were higher with invalid than valid retro-cues (21 vs. 17), $F(1, 24) = 9.87$, $p = .004$, $\eta_p^2 = .291$. The interaction between cue reliability and validity was not significant, $F(1, 24) = 1.75$, $p = .198$, $\eta_p^2 = .068$. Mean *SD* was 18.2.

Experiment 2

Swap Model. For guess rates, and similar to the results of Experiment 1, there were no main effects of retro-cue reliability, $F(1, 24) = 0.02$, $p = .878$, $\eta_p^2 = .001$, validity, $F(1, 24) < 0.01$, $p = .983$, $\eta_p^2 < .001$, and no interaction between the retro-cue reliability and validity, $F(1, 24) = 0.17$, $p = .687$, $\eta_p^2 = .007$. Mean guess rate was .290.

For swap rates, there was no main effect of retro-cue reliability, $F(1, 24) = 3.58$, $p = .071$, $\eta_p^2 = .130$ and, different from Experiment 1, no main effect of validity either, $F(1, 24) = 4.01$, $p = .057$, $\eta_p^2 = .143$. The interaction between the retro-cue reliability and validity was also not significant, $F(1, 24) = 2.10$, $p = .161$, $\eta_p^2 = .080$. Mean swap rate was .310. Taken together with the guess rate (.290), this suggests that approximately 60% of responses did not correspond to the target item, either due to random guessing or confusions with non-target items.

Concerning the *SD*, there were no effects of retro-cue reliability, $F(1, 24) = 0.37$, $p = .547$, $\eta_p^2 = .015$, or validity, $F(1, 24) = 0.06$, $p = .804$, $\eta_p^2 = .003$. However, the interaction between cue reliability and validity was significant, $F(1, 24) = 6.03$, $p = .022$, $\eta_p^2 = .201$. However, the difference between valid and invalid trials was not significant at 33% retro-cue

reliability (24.9 vs. 25.7), $t(24) = –0.91$, $p_{holm} = .371$, $d_z = –0.18$ [–0.56, 0.21], nor at 70% cue reliability (26.1 vs. 25.1), $t(24) = 1.90$, $p_{holm} = .130$, $d_z = 0.37$ [–0.02, 0.76].

Experiment 3

Swap Model. For guess rates, there was no main effect of the retro-cue reliability, $F(1, 23) = 0.75$, $p = .394$, $\eta_p^2 = .032$, and no interaction between the retro-cue reliability and validity, $F(1, 23) = 0.41$, $p = .527$, $\eta_p^2 = .018$. However, contrary to the previous two experiments, there was a main effect of validity, $F(1, 23) = 21.53$, $p < .001$, $\eta_p^2 = .484$, indicating larger guess rates for invalid than valid retro-cues (.120 vs .040). The mean guess rate was .08.

For swap rates, there was no main effect of retro-cue reliability, $F(1, 23) = 0.90$, $p = .353$, $\eta_p^2 = .038$, and the interaction between cue reliability and cue validity, $F(1, 23) = 3.76$, $p = .065$, $\eta_p^2 = .141$ was not significant either. Similar to Experiment 1, the main effect of validity was significant, $F(1, 23) = 6.84$, $p = .016$, $\eta_p^2 = .229$, suggesting larger p_{swap} for invalid than valid retro-cues (.04 vs .02). The mean swap rate was .03. Taken together with the mean guess rate (.08), this indicates that approximately 11% of responses were not attributed to the target item, either due to random guessing or confusion with a non-target.

For *SD*s, results again converge with those of Experiment 1 in that there was no effect of retro-cue reliability, $F(1, 23) = 3.52$, $p = .073$, $\eta_p^2 = .133$, but a main effect of cue validity, $F(1, 23) = 28.55$, $p < .001$, $\eta_p^2 = .554$, and an interaction between the cue reliability and cue validity, $F(1, 23) = 10.03$, $p = .004$, $\eta_p^2 = .304$. For the 50% cue reliability, invalid trials had a higher *SD* than valid trials (19 vs. 17), $t(23) = 2.55$, $p_{holm} = .018$, $d_z = 0.52$ [0.09, 0.95], and this difference increased with 70% cue reliability (22 vs. 16), $t(23) = 5.29$, $p_{holm} < .001$, $d_z = 1.08$ [0.58, 1.58].

Comparison of rejection

In Table 1, for each analysis, we applied our exclusion criteria to identify outliers. However, following Miller's (2023) observation that outliers alone can alter or skew results, we also provide a table in the supplementary material showing participants' performance with and without exclusions. In Table 1, we compare the ANOVA results from Experiments 1, 2, and 3 reported in the paper with those obtained without exclusions (the "noRej" analysis).

As indicated in the paper, for all experiments, in the search task we excluded RTs smaller than 200 ms and larger than 2000 ms. Only correct trials were retained. We then removed trials with RTs deviating by more than 2.5 *SD*s from the condition mean (computed on all trials). In the "noRej" analysis, we include all the correct responses.

For the RTs memory task, as reported in the manuscript, we retained only RTs between 500 and 4000 ms. In the "noRej" analysis, we included all values.

One final difference concerns Experiment 1. As stated in the manuscript, we removed one participant (Participant 16) due to his guess rate. In the "noRej" analysis, we decided to include him.

Table S1. *Comparison of results with and without outlier exclusions.*

**Experiment 1**						
**ANOVA RT Search Task**						
	F main	*F* noRej	*p* main	*p* noRej	η_p^2 main	η_p^2 noRej
Cue validity	25.61	29.37	< .001	< .001	.516	0.540
Cue reliability	0.50	1.02	.488	.323	.020	.039
Cue validity × reliability	7.30	2.86	.012	**.103**	.233	.103
**Post hoc RT Search Task**						
	t main	*t* noRej	*p* main	*p noRej*	d_z main	d_z noRej
Invalid vs. valid at 50% Cue reliability	4.70	4.53	< .001	< .001	–0.94 [–0.44, –1.44]	0.89 [0.41, 1.37]
Invalid vs. valid at 70% Cue reliability	4.91	5.19	< .001	< .001	–0.98 [–0.48, –1.49]	1.02 [–0.52, –1.52]
50% vs. 70% Cue reliability within invalid trials	–1.72	–1.29	.196	.416	–0.34 [–0.77, 0.08]	–0.25 [–0.66, 0.16]
50% vs. 70% Cue reliability within valid trials	0.52	–0.42	.606	.680	0.10 [–0.31, 0.52]	–0.08[–0.49, 0.32]
**Recall Error Memory Task**						
	F main	*F* noRej	*p* main	*p* noRej	η_p^2 main	η_p^2 noRej
Cue validity	17.51	11.97	< .001	< .001	.422	.324
Cue reliability	0.94	2.02	.342	.168	.038	.075
Cue validity × reliability	0.05	0.33	.826	.571	.002	.013
**Accuracy Search Task**						
	F main	*F* noRej	*p* main	*p* noRej	η_p^2 main	η_p^2 noRej
Cue validity	10.18	11.26	< .001	< .001	.298	.311
Cue reliability	0.34	0.13	.566	.717	.014	.005
Cue validity × reliability	0.60	0.82	.445	.375	.025	.032
**RT Memory Task**						
	F main	*F* noRej	*p* main	*p* noRej	η_p^2 main	η_p^2 noRej
Cue validity	18.13	13.37	< .001	< .001	.430	.348
Cue reliability	0.11	1.70	.746	.205	.004	.064
Cue validity × reliability	5.14	2.52	.033	**.125**	.177	.091
**Post hoc RT Memory Task**						
	t main	*t* noRej	*p* main	*p* noRej	d_z main	d_z noRej
50% Cue reliability	1.94	1.72	.065	.099	0.37 [–0.02, 0,77]	0.33 [–0.06, 0.71]
70% Cue reliability	5.43	3.79	< .001	< .001	1.05 [0.57, 1.53]	0.72 [0.30, 1.14]
**Experiment 2**						
**RT Search Task**						
	F main	*F* noRej	*p* main	*p* noRej	η_p^2 main	η_p^2 noRej
Cue validity	85.04	73.05	< .001	< .001	.780	.753
Cue reliability	0.59	0.45	.450	.508	.024	.019

Cue validity × reliability	27.25	14.29	< .001	< .001	.532	.373
**Post hoc RT Search Task**						
	t main	*t* noRej	*p* main	*p* noRej	d_z main	d_z noRej
Invalid vs. valid at 33% Cue reliability	6.73	6.02	< .001	< .001	1.35 [0.77, 1.92]	1.2 [0.66, 1.75]
Invalid vs. valid at 70% Cue reliability	9.08	8.15	< .001	< .001	1.82 [1.14, 2.50]	1.63 [0.99, 2.27]
70% vs. 33% cue reliability within invalid trials	3.56	2.77	.002	.018	0.71 [0.25, 1.18]	0.55 [0.11, 1.00]
33% vs.70% cue reliability within valid trials	4.44	2.84	< .001	.018	0.89 [0.40, 1.38]	0.57 [0.12, 1.01]
**Accuracy Search Task**						
	F main	*F* noRej	*p* main	*p* noRej	η^2_p main	η^2_p noRej
Cue validity	102.91	89.26	< .001	< .001	.811	.788
Cue reliability	1.06	1.70	.31	.205	.042	.066
Cue validity × reliability	20.06	20.18	< .001	< .001	.455	.457
**Post hoc Accuracy Search Task**						
	t main	*t* noRej	*p* main	*p* noRej	d_z main	d_z noRej
33% Cue reliability	–5.47	–4.92	< .001	< .001	–1.09[–1.59, –0.60]	–0.98 [–1.46, –0.51]
70% Cue reliability	–9.89	–9.56	< .001	< .001	–1.98 [–2.65, –1.30]	–1.91 [–2.57, –1.25]
**RT Memory Task**						
	F main	*F* noRej	*p* main	*p* noRej	η^2_p main	η^2_p noRej
Cue validity	141.78	62.91	< .001	< .001	.855	.724
Cue reliability	2.99	0.42	.100	.522	.111	.017
Cue validity × reliability	32.66	10.81	< .001	< .001	.576	.311
**Post hoc RT Memory Task**						
	t main	*t* noRej	*p* main	*p* noRej	d_z main	d_z noRej
33% Cue reliability	6.80	2.45	< .001	.022	1.36 [0.82, 1.90]	0.49 [0.08, 0.91]
70% Cue reliability	11.99	10.35	< .001	< .001	2.40 [1.63, 3.17]	2.07 [1.38, 2.77]
**Experiment 3**						
**RT Search Task**						
	F main	*F* noRej	*p* main	*p* noRej	η^2_p main	η^2_p noRej
Cue validity	110.41	89.35	< .001	< .001	.828	.795
Cue reliability	10.24	0.73	.004	**.401**	.308	.031
Cue validity × reliability	65.37	45.23	< .001	< .001	.740	.663
**Post hoc RT Search Task**						
	t main	*t* noRej	*p* main	*p* noRej	d_z main	d_z noRej
Invalid vs. valid at 50% Cue reliability	7.85	7.51	< .001	< .001	1.6 [0.96, 2.25]	1.53 [0.90, 2.16]
Invalid vs. valid at 70% Cue reliability	10.46	9.28	< .001	< .001	2.13 [1.36, 2.91]	1.89 [1.18, 2.61]
70% vs. 50% cue reliability within invalid trials	6.29	3.13	< .001	.009	1.28 [0.71, 1.86]	0.64 [0.17, 1.10]

50% vs. 70% cue reliability within valid trials	3.77	3.11	.001	.009	0.77 [0.29, 1.25]	0.63 [0.17, 1.10]
**Accuracy Search Task**						
	F main	*F* noRej	*p* main	*p* noRej	η_p^2 main	η_p^2 noRej
Cue validity	47.27	47.27	< .001	< .001	.673	.673
Cue reliability	7.74	4.99	.011	.036	.252	.178
Cue validity × reliability	27.10	23.20	< .001	< .001	.541	.502
**Post hoc Recall Accuracy Search Task**						
	t main	*t* noRej	*p* main	*p* noRej	d_z main	d_z noRej
50% Cue reliability	–5.38	–5.45	< .001	< .001	–1.10 [–1.61, –0.59]	–1.11 [–1.62, –0.60]
70% Cue reliability	–7.35	–7.22	< .001	< .001	–1.50 [–2.08, –0.92]	–1.47 [–2.05, –0.90]
**RT Memory Task**						
	F main	*F* noRej	*p* main	*p* noRej	η_p^2 main	η_p^2 noRej
Cue validity	58.76	57.76	< .001	< .001	.719	.715
Cue reliability	0.57	1.34	.459	.260	.024	.055
Cue validity × reliability	44.02	45.06	< .001	< .001	.657	.662
**Post hoc RT Memory Task**						
	t main	*t* noRej	*p* main	*p* noRej	d_z main	d_z noRej
50% Cue reliability	4.09	5.00	< .001	< .001	0.84 [0.37, 1.30]	1.02 [0.53, 1.51]
70% Cue reliability	8.43	8.16	< .001	< .001	1.72 [1.09, 2.35]	1.67 [1.05, 2.28]

Note. noRej = no rejection. For Experiment 1, analyses include one extra participant when outliers are not rejected. *P* values in **bold red** indicate that the statistical significance of the effect changed when the outliers were excluded.

Supplementary references

Bays, P. M. (2016). Evaluating and excluding swap errors in analogue tests of working memory. *Scientific Reports*, *6*(1), 19203. https://doi.org/10.1038/srep19203

Bays, P. M., Catalao, R. F. G., & Husain, M. (2009). The precision of visual working memory is set by allocation of a shared resource. *Journal of Vision*, *9*(10), 7. https://doi.org/10.1167/9.10.7

Miller, J. (2023). Outlier exclusion procedures for reaction time analysis: The cures are generally worse than the disease. *Journal of Experimental Psychology. General*, *152*. https://doi.org/10.1037/xge0001450

Suchow, J. W., Brady, T. F., Fougnie, D., & Alvarez, G. A. (2013). Modeling visual working memory with the MemToolbox. *Journal of Vision*, *13*(10), 9. https://doi.org/10.1167/13.10.9

Williams, J. R., Brady, T. F., & Störmer, V. S. (2022). Guidance of attention by working memory is a matter of representational fidelity. *Journal of Experimental Psychology: Human Perception and Performance*, *48*(3), 202–231. https://doi.org/10.1037/xhp0000985

Williams, J. R., Robinson, M. M., & Brady, T. F. (2023). There is no theory-free measure of "swaps" in visual working memory experiments. *Computational Brain & Behavior*, *6*(2), 159–171. https://doi.org/10.1007/s42113-022-00150-5
